# Novel Nanocomposites Based on Bacterial Polyester/LDH-SDS Clay for Stem Cells Delivery in Modern Wound Healing Management

**DOI:** 10.3390/ma13204488

**Published:** 2020-10-10

**Authors:** Eugeniu Vasile, Ionut-Cristian Radu, Bianca Galateanu, Maria Rapa, Ariana Hudita, Dana Jianu, Paul-Octavian Stanescu, Horia Cioflan, Catalin Zaharia

**Affiliations:** 1 Faculty of Applied Chemistry and Materials Science, Politehnica University of Bucharest, 060042 Bucharest, Romania; eugeniuvasile@yahoo.com; 2 Advanced Polymer Materials Group, Politehnica University of Bucharest, 060042 Bucharest, Romania; radu.ionucristian@gmail.com (I.-C.R.); paul_stanescu@yahoo.com (P.-O.S.); 3 Department of Biochemistry and Molecular Biology, University of Bucharest, 91-95 Splaiul Independentei Street, 050095 Bucharest, Romania; bianca.galateanu@bio.unibuc.ro (B.G.); arianahudita@yahoo.com (A.H.); 4 ECOMET Center, Politehnica University of Bucharest, 313 Splaiul Independentei, 060042 Bucharest, Romania; rapa_m2002@yahoo.com; 5 International Society of Regenerative Medicine and Surgery, 38-40 T. Stefan Street, 011658 Bucharest, Romania; zhctln@yahoo.com; 6 SANADOR Clinic Hospital, Sevastopol Street, No. 9, Sector 1, 010991 Bucharest, Romania; cioflanirina@yahoo.com; 7 Department of General Surgery, Oncology Institute “Prof.dr. Alexandru Trestioreanu”, Sos. Fundeni, Bucharest, No. 252, Sector 2, 022328 Bucharest, Romania

**Keywords:** polyhydroxyalkanoates, LDH-SDS, nanocomposite, melt processing, tissue engineering

## Abstract

Nanocomposite materials based on poly(3-hydroxybutyrate-co-3-hydroxyvalerate) (PHBHV) and modified mineral clay layered double hydroxides (LDH-SDS) were explored as novel nanostructured materials for potential tissue engineering applications. The mineral clay inorganic phase was modified with an anionic long-chain structure of carbon atoms, such as sodium dodecyl sulfate, in order to increase the compatibility between the two phases. The melt intercalation method used for nanocomposite fabrication ensures a good dispersion of the modified LDH-SDS within the polymer matrix without using a toxic solvent (chloroform). The nanocomposites were found to have an intercalated/exfoliated structure with an enhanced Young modulus and increased stiffness. This could allow them to be considered for autologous stem cells dressings in the view of efficient wound healing applications.

## 1. Introduction

Polyhydroyalkanoates (PHAs) represent a versatile category of naturally occurring biodegradable polyesters from renewable sources [1]. In recent years, they have represented a good alternative to non-biodegradable polymers because they are biopolymers, in which the structural units are bonded by the ester linkage. These types of ester linkages can be found in nature as well as in esterase enzymes, which can recognize and degrade them [2,3]. Normally, the biodegradability of these biopolymers is achieved by decomposition in biological conditions through enzymes produced by living microorganisms like bacteria. In contrast to petroleum-derived polymers, PHAs are fully biodegradable biopolymers with zero toxic waste [4,5,6].

Poly(3-hydroxybutyrate-co-3-hydroxyvalerate) (PHBHV) is one of the most common members of the poly(β-hydroxyalkanoate)s family. PHBHV is a copolymer of poly(3-hydroxybutyrate) (PHB), which is produced via a fermentative process where propionic acid and glucose (carbon source) are added [7]. PHBHV was developed to improve the mechanical properties of PHB, namely its low thermal stability, brittleness, and narrow process window for industrial application. The stiffness and brittleness of PHB are as a result of its high crystallinity and isotactic structure. Introduction of 3-hydroxyvalerate (3HV) units decreases the specific temperatures, glass transition, melting temperatures, and crystallinity, improving it for industrial processing. The copolymer needs a better impact resistance, toughness, and flexibility for industrial applications, which is mostly achieved by melt processing, such as injection and extrusion molding [8,9]. The co-monomer ratio between 3-hydroxybutyrate (3HB) and 3-hydroxyvalerate (3HV) units depends on the ratio of propionic acid to glucose, resulting a random copolymer [10,11].

The biodegradability, biocompatibility, mechanical strength, and the ability to support cell attachment and proliferation are of great interest for the production of fast usage products and for medical application, for example as tissue engineering scaffolds for tissue engineering, and for the repair of cartilage and bone [12,13,14].

In the last decades, interest in the applications of nanocomposite materials increased as the matrix–nanofiller formulation gives enhanced properties compared with the classical composites [15]. Clay minerals are very extended compounds with growing interest for use as nanocomposite materials as they can replace traditional fillers. This ability is directly related to their size; crystalline structure; and properties, namely, a high specific surface area, good rheological properties, and good adsorptive capacity.

Hydrotalcite is a natural hydroxycarbonate of Mg and Al with a hexagonal structure, where magnesium cations fill the holes between every two layers, which are known as layered double hydroxides (LDHs) [16,17,18]. The substitution of magnesium cations (Mg^2+^) with aluminum cations (Al^3+^) causes positive charges between the layers, and these positive charged layers are compensated by anions found between the layers, whose chemical formula is given by the general formula [MII1 − xMIIIx(OH)_2_]x + [An−x/n·yH_2_O]x−, where MII and MIII are divalent and trivalent metal cations, An− is an n-valent anion, and x has values usually between 0.20 and 0.33 [19,20]. Layered double hydroxides are compounds with a biological potential and allow for the intercalation of other molecules between their layers, opening their application in medicine and pharmacy [21,22]. In contrast to classic fillers, the layered structure allows for the intercalation of the polymer chains between layers with the formation of an intercalated or exfoliated structure. The strong attractive forces between the layers and layered structure enhance the mechanical properties and composite performance [23,24]. The physical attraction forces occurring between the organic phase (polymer) and inorganic phase (mineral clay) can be improved through the modification of LDH with several types of organic species. The modification supposes the substitution of small inorganic anions between layers with larger organic anions with a hydrocarbon long chain (dodecyl sulfate, benzenesulfonate, and laurate and bis(2-ethylhexyl)hydrogen phosphate) [25,26,27].

Since the earliest days of civilization, clays have been used for medicinal purposes [28,29,30] to purify the blood and to treat infection and ulcers [31,32]. Nanocomposites based on polymers and clay have gained great attention as a new composite material family that exhibits exceptional improvements for the material properties as compared with the matrix polymers. The applicability area of the polymer–clay nanocomposite biomaterials could be expanded towards use in the design of new drug-release devices with specific technological and biopharmaceutical properties, such as swelling, film forming, bio-adhesion, and cell uptake [33].

The latest strategies in the current tissue engineering applications consist of the design of biocomposites obtained by pre-seeding smart biomaterials with stem cells in order to heal or regenerate damaged tissues. Zuk et al. have shown that subcutaneous adipose tissue is an advantageous mesenchymal-like stem cell source because of its abundancy, accessibility, minimal morbidity, and the discomfort associated with its harvest [34]. Human adipose stem cells (hASCs) secrete many of the growth factors that take part in the normal wound healing process [35,36,37], and after delivery, these cells may remain viable and secrete growth factors in a continuous manner, as this occurs in vivo in the normal wound healing process.

In this study, novel nanocomposites have been fabricated by melt intercalation of poly(3-hydroxybutyrate-co-3-hydroxyvalerate) and LDH clay modified with sodium dodecyl sulfate (PHBHV/LDH-SDS) as possible autologous stem cells dressings for prospective wound healing applications.

## 2. Materials and Methods

### 2.1. Materials

Poly(3-hydroxybutyrate-co-3-hydroxyvalerate) (PHBHV) granules (12% HV) were supplied from Good Fellow Cambridge Limited (Huntingdon PE29 6WR, UK). Dodecyl sodium sulfate was provided by Sigma Aldrich. The modified mineral clay-like layered double hydroxide with dodecyl sodium sulfate (LDH-SDS) was obtained through the co-precipitation method. A solution of Mg(NO_3_)_2_∙6H_2_O (9.6 g) and Al(NO_3_)_3_∙9H_2_O (4.7 g) in H_2_O (45 mL) was obtained. Another solution was obtained by dissolving SDS (4 g) and NaOH (3.4 g) in 50 mL of H_2_O under stirring. The pH value was kept at around 10 and the mixture was aged for 22 h at 80 °C. The precipitate was filtered and washed with 2 L of hot water. Finally, the precipitate was dried for 48 h at 80 °C.

### 2.2. Methods

#### 2.2.1. Fabrication of PHBHV/LDH-SDS Plates

**Figure 1:**
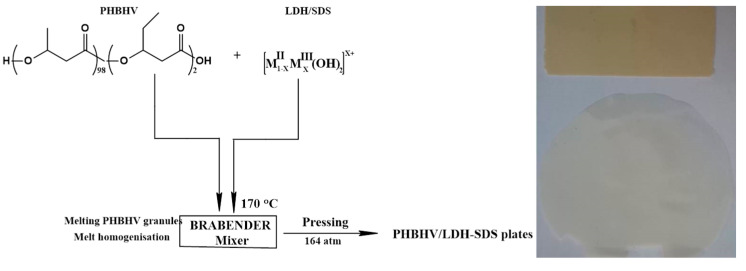
Obtaining polymer–clay nanocomposites by melt pressing**.**

The composite materials were prepared by melt mixing poly (3-hydroxybutyrate-co-3-hydroxyvalerate) and modified mineral clay (LDH-SDS) at 170 °C on a Brabender device for 6 min, with 50 rpm rotation. The obtained melt compositions were subjected to P 200P Colin 164 laboratory press for 2 min at 164 atm and a suitable temperature. Plate materials with different ratios of PHBHV/LDH-SDS (0, 1, 3, and 5% *w*/*w*) were obtained (Figure 1).

#### 2.2.2. Characterization

X-ray diffraction analysis: PHBHV/LDH-SDS nanocomposites obtained through the melt intercalation method were characterized by X-ray diffraction (XRD). The XRD patterns were registered on a Panalytical X’PERT MPD Power Diffractometer (PANalytical, Almelo, The Netherlands) in the range of 2θ = 10–80°. An X-ray beam characteristic of Cu Kα radiation was used (λ = 1.5418 Å).

Tensile strength: The nanocomposite plates were cut in a milling process, and specimens with 10 mm width and 1 mm thickness for tensile test were obtained. An Instron 2519-107 Universal test machine (Instron^®^, Norwood, MA, 02062-2643, USA) with a 2 mm/min speed and 5 kN load cell was used to measure the mechanical properties (Young modulus, tensile strength, and elongation at break) at room temperature.

Atomic force microscopy (AFM): The non-contact (tapping) mode AFM images were obtained using multimode Agilent 5500 (Agilent Technologies, Santa Clara, CA, USA) apparatus. The atomic force microscope was equipped with an AC Mode III controller and with a scanner with a maximum x/y scan range of 90 µm × 90 µm and a z scan range of 7 µm. The analyses were carried out at room temperature using a typical silicon nitride non-contact cantilever with a spring constant of 48 N/m, a nominal tip radius of 10 nm, and were operated at resonant frequency of 190 kHz (Agilrom).

Transmission electron microscopy (TEM): The morphological analysis, including the structure of the crystalline phase of the PHBHV/LDH-SDS nanocomposites, was investigated through high-resolution transmission electron microscopy (HR-TEM) using a TECNAI G^2^ F30 S-TWIN microscope (FEI-Philips, Hillsboro, OR, USA) operated at 300 kV with an energy dispersive X-ray analysis (EDAX) facility.

#### 2.2.3. Cell Culture Model

Human adipose derived stem cells (hASCs) were employed for the in vitro assessment of the PHBHV, 1% PHBHV/LDH-SDS, 3% PHBHV/LDH-SDS, and 5% PHBHV/LDH-SDS nanocomposites’ biocompatibility.

The human subcutaneous adipose tissue was harvested from female patients partaking in elective liposuction. All medical procedures were performed in the Proestetica Medical Center and were conducted in compliance with the Helsinki Declaration, with the approval of the Ethical Committee of the Proestetica Medical Center (reference number 112/23.10.2014). All patients were in good health and provided written consent approving their participation (confidential).

The hASCs were isolated from the human subcutaneous adipose tissue, as described by the authors of [38], and the obtained culture was validated through the investigation of the mesenchymal stem cells’ surface markers panel through flow cytometry [39].

##### Fluorescent Labeling of Actin Filaments

The protein expression of actin was studied 24-h post seeding through confocal fluorescence microscopy using a Carl Zeiss LSM710 confocal microscope (Carl Zeiss Microscopy GmbH, Jena, Germany). The nanobiohybrids were fixed with 4% PFA for 1h and permeabilized with 2% BSA/0.1% Triton X-100 solution at 4 °C. The constructs were then incubated overnight at 37 °C with a Phalloidin-Alexa Fluor 546. The next day, cells’ nuclei were stained for 30 min with DAPI (4′,6-diamidino-2-phenylindole) and the samples were inspected with confocal microscopy (Carl Zeiss Zen 2010 software 6.0).

##### MTT Assay

The hASCs’ viability when in contact with the nanocomposite materials was quantitatively determined using the MTT spectrophotometric assay at 24 h post seeding. In this context, all of the cell-bioconstructs were incubated in a 1 mg/mL MTT (Thiazolyl Blue Tetrazolium Bromide) solution for 4 h. The formazan crystals that were obtained were dissolved in isopropanol for 30 min at room temperature. The absorbance of the resulting solution was measured by spectrophotometry at 550 nm (AppliskanThermo Scientific, Waltham, MA, USA).

##### Lactate Dehydrogenase Assay

The cytotoxic potential of the nanocomposites on the hASCs was evaluated using the “In vitro toxicology assay kit lactate dehydrogenase-based” kit according to the manufacturer’s protocol. Briefly, the culture media were harvested at 24 h post seeding and were mixed with the solutions provided in the kit. After 20 min of incubation at room temperature and darkness, the lactate dehydrogenase activity was determined by measuring the optic density of the resulting solutions at 490 nm.

##### Statistical Analysis

GraphPad Prism 3.03 Software (San Diego, CA, USA) was used for the spectrophotometric data analysis and processing. One-way ANOVA and the Bonferroni test were applied on the raw data. All experiments were performed in biological triplicates, and each data set was presented as the average of three replicates (mean ± standard deviation (SD)).

## 3. Results and Discussions

### 3.1. X-ray Diffraction

One of the most important studies to reveal the dispersion of modified mineral clay and the exfoliation mechanism is X-ray diffraction analysis. XRD shows the diffraction plans and changes of the interlayer distance, which appear in the mineral clay. The modification of mineral clay with the anionic long-chain structure of carbon atoms, such as sodium dodecyl sulfate, has increased the compatibility between the two phases, namely, an organic phase represented by polyester and an inorganic phase represented by mineral clay type layered double hydroxides.

**Figure 2:**
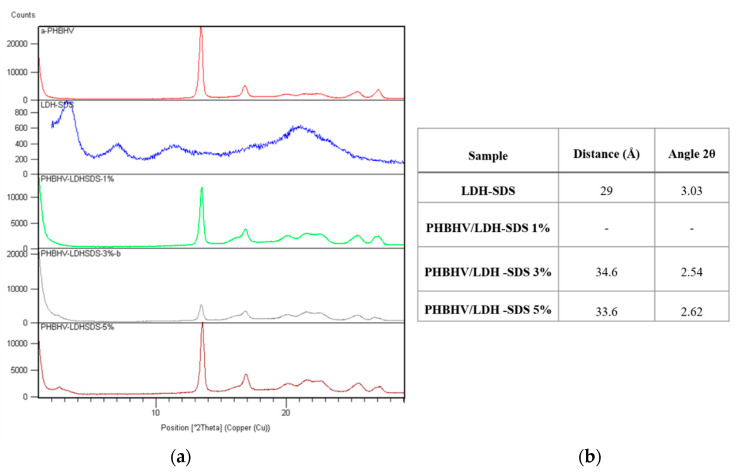
(**a**) XRD patterns of modified mineral clay (LDH-SDS), Poly(3-hydroxybutyrate-co-3-hydroxyvalerate) (PHBHV), and (**b**) PHBHV/LDH-SDS nanocomposites (1, 3, and 5%).

At the same time, the sodium dodecyl sulfate is a bigger anion, and it replaces interlayer inorganic anions, leading to an increase in the interlayer distance. In Figure 2, we show that the crystalline phase of the modified clay has a specific peak at an angle of 2θ 3.03°.

For the nanocomposites with 3% and 5% LDH-SDS, the specific peak is shifted to lower 2θ values, namely 2θ 2.54° and 2θ 2.62°. The decrease in 2θ values leads to an increase in the interlayer distance and suggests the penetration of polyester chains between clay layers. This behavior is supported by the modification of clay with sodium dodecyl sulfate. This has a carbon atom chain structure favoring the formation of physical bonds between the organic anion and polymer.

The composition with 1% LDH-SDS does not show this diffraction peak, suggesting that the orderly arrangement of clay diffraction layers is perturbed by the introduction of the natural polyester. This leads to intercalated nanocomposites, where an ordered multilayered structure is obtained or even exfoliated nanocomposites, where the clay layers are completely and uniformly dispersed within the polymer matrix.

Most probably the highest content of LDH/SDS (5%) supposes a higher distribution within the polymer matrix because of the interactions between the clay particles. In higher amounts, clay particles are able to interact with each other and not only with the polymer chains. This fact probably increases the compatibility degree.

### 3.2. Tensile Strength

**Figure 3:**
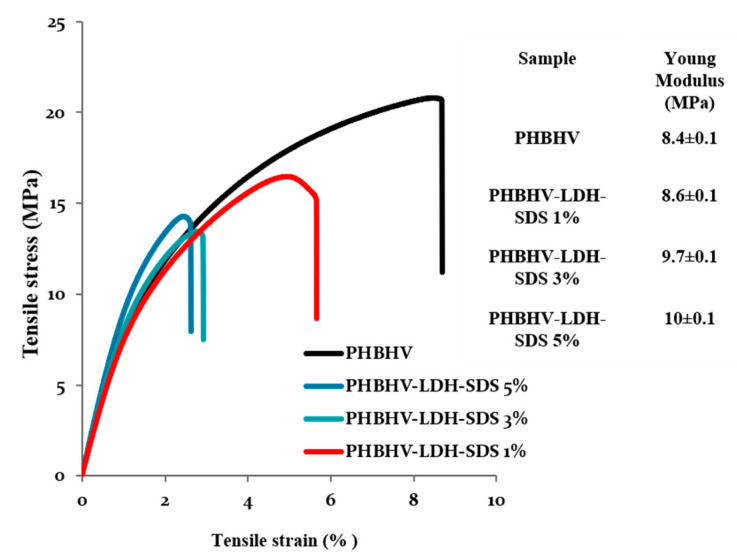
Tensile stress versus tensile strain for PHBHV and PHBHV/LDH-SDS nanocomposites**.**

The tensile strength test confirms the rigidity of the poly (3-hydroxybutyrate-3-hydroxyvalerate) with elongation at a break of 8% and an elastic modulus of 8.4 MPa (Figure 3). The composite materials obtained after dispersion had a lower elongation at break and showed an increase in the Young modulus after increasing the amount of mineral clay. These results showed a direct action of mineral clay on the mechanical properties of composite materials resulting in increased stiffness. Adding small amounts of additives (citric esters) can change the useful properties by decreasing the tensile strength and Young’s modulus.

### 3.3. Atomic Force Microscopy (AFM)

**Figure 4:**
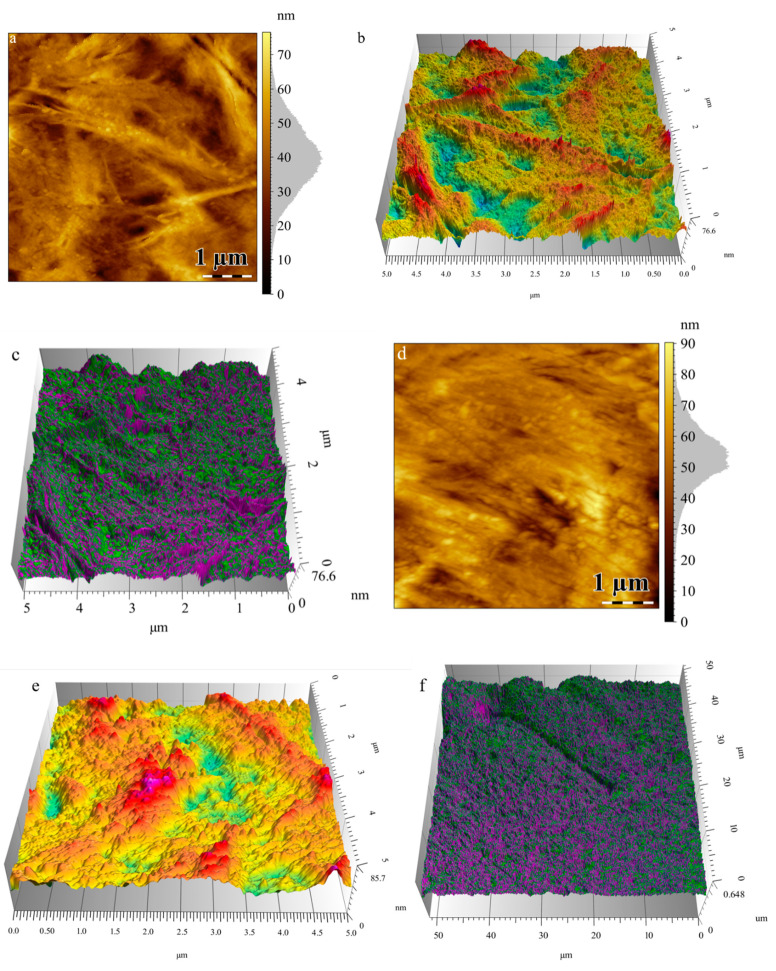
Atomic force microscopy (AFM) analysis: (**a**) Topography PHBHV/LDH-SDS 5%, (**b**) 3D image PHBHV/LDH-SDS 5%, (**c**) high magnification phase image PHBHV/LDH-SDS 5%, (**d**) topography PHBHV, (**e**) 3D image PHBHV, and (**f**) low magnification phase image PHBHV/LDH-SDS 5%. Color code: Topography (bright—high areas; dark—low areas); phase distribution (green—clay; mauve—polymer matrix)**.**

Figure 4 shows the height images and 3D topography of PHBHV/LDH-SDS 5% nanocomposite plates and PHBHV plates without mineral clay. The height images reveal the morphology of the sample and the modification of the surface structure, which appears with the mineral clay introduction. The natural polyester PHBHV shows a structural arrangement with higher features, is less rigid, and tends to adhere to cantilever tip.

**Figure 5:**
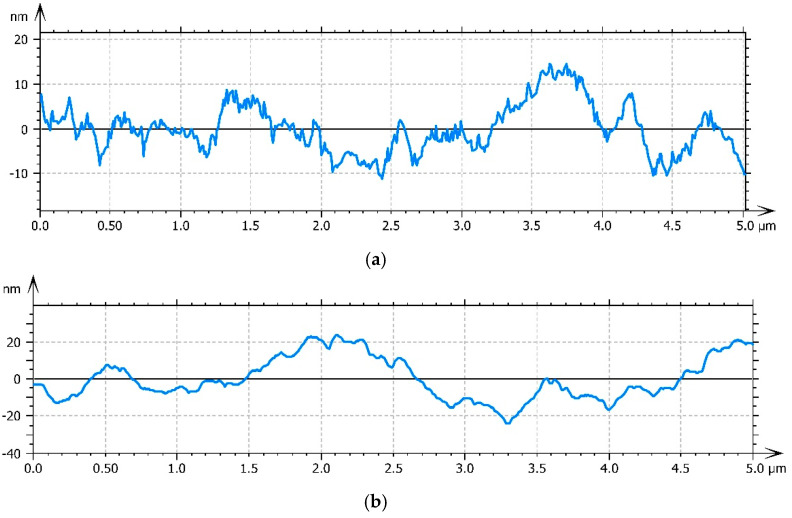
Roughness profiles: PHBHV (**a**) and PHBHV/LDH-SDS 5% (**b**).

The nanocomposites with 5% modified mineral clay show a topography with smaller features and a decrease in roughness (Figure 5). The arithmetic average roughness (Ra) was measured by roughness profiles using ISO 4287:1997 [40] in several areas of the surface. The decrease in the arithmetic average roughness from 6.31 nm for PHBHV to 3.55 nm for nanocomposites with 5% LDH-SDS is shown by the different structural arrangement of the sample’s surface.

This imaging mode is an extension of the tapping mode, where a relationship between the structure and properties of the material can be seen. In the tapping mode, the cantilever oscillates at or near its resonance frequencies, and if some variations appear in the amplitude oscillation, the adhesion behavior and energy dissipation vary, also resulting in a contrasted image. In the high and low magnification phase images, a good distribution of the modified mineral clay on the polyester surface with a better compatibility between organic and inorganic phases can be seen. The topography images reveal modification upon the clay addition. This fact is supported by clear changes in the roughness profile and phase distribution imaging. The addition of clay likely led to a more compact structuring.

### 3.4. Transmission Electron Microscopy (TEM)

**Figure 6:**
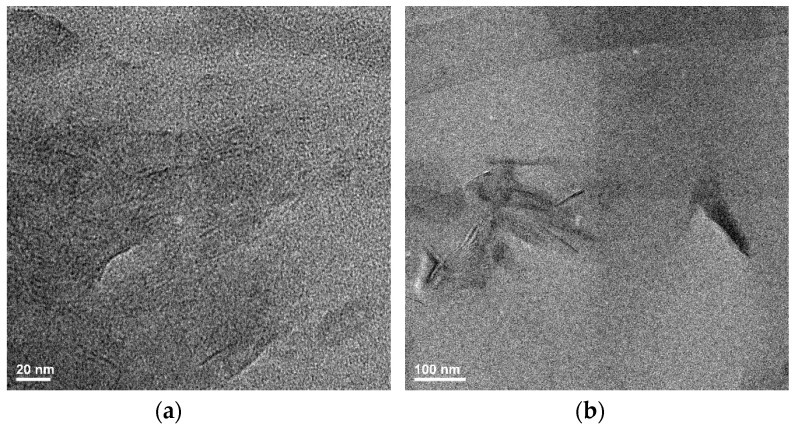
Transmission electron microscopy (TEM) images for the PHBHV/LDH-SDS 5% nanocomposite. (**a**) high magnification 20 nm; (**b**) low magnification 100 nm.

**Figure 7:**
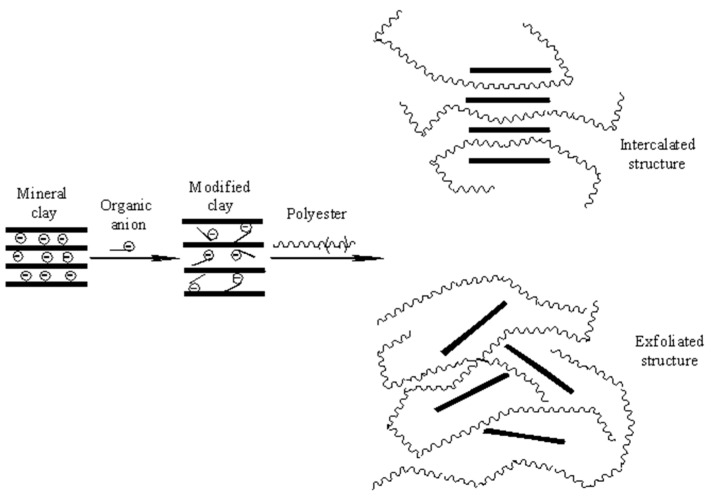
Intercalation and exfoliation mechanism**.**

TEM analysis was employed to support the XRD results and the idea of obtaining intercalated and exfoliated structures (Figure 6). Layered double hydroxides are layered clays with water molecules and anions between the layers in order to compensate for the positive charge of the layers. The penetration of the polyester macromolecules between the clay layers leads to an increase in interlayer distance, obtaining a multi-layered intercalated structure where the polyester-clay layers alternate (Figure 7). In the case of well dispersed clay layers in a polymer matrix, an exfoliated structure is obtained where the layers are no longer oriented in one direction (Figure 7). For the nanocomposites with 1% modified mineral clay (PHBHV/LDH-SDS 1%), the X-ray diffraction results show the absence of any specific diffraction peak at the angle 2θ 3.03°, leading to perturbed clay layers and obtaining intercalated and exfoliated structures. These results are not supported by the TEM results and demonstrate that no intercalated or exfoliated formed. The X-ray diffraction results for PHBHV/LDH-SDS 3% show an increase in the interlayer distance, but these findings are not supported by the TEM analysis. For the nanocomposites with 5% modified mineral clay, the X-ray diffraction results are supported by the TEM images. In Figure 6, the low magnification TEM image shows the clay particles dispersed in a polyester matrix without large aggregates being observed. The high magnification image reveals a disorderly orientation of clay layers in a polymer matrix, obtaining an intercalated and exfoliated structure. The TEM results prove that a larger amount of mineral clay may lead to a better dispersion of the clay particles and to the formation of intercalated/exfoliated nanocomposite structures.

### 3.5. hASCs Morphology on PHBHV, PHBHV/LDH-SDS 1%, PHBHV/LDH-SDS 3%, and PHBHV/LDH-SDS 5% Biomaterials

**Figure 8:**
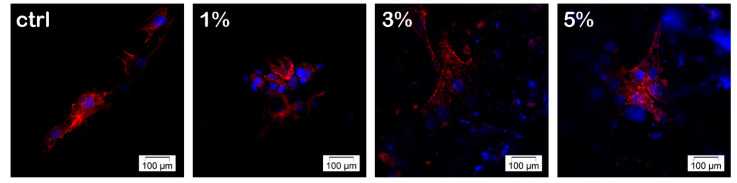
Confocal fluorescence microscopy micrographs of human adipose stem cells’ (hASCs) actin filaments networks (red fluorescence) in hASCs/PHBHV, hASCs/PHBHV/LDH-SDS 1%, hASCs/PHBHV/LDH-SDS 3%, and hASCs/PHBHV/LDH-SDS 5% nanocomposites. The DAPI-stained nuclei are blue.

The hASC’s morphology and the ability to interact with a material in terms of adhesion and cytoskeleton development were investigated after 24 h of hASCs culture on nanocomposite biomaterials. The hASCs from all of the biohybrids displayed long and distinctive actin filaments surrounding the nuclei, which clearly determined the overall cell morphology (Figure 8).

**Figure 9:**
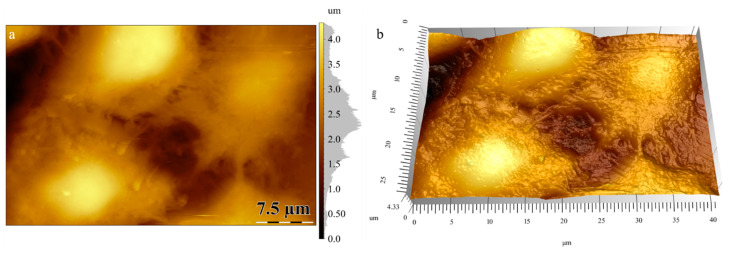
Topography 2D (**a**) and 3D (**b**) images for osteoblast cell line adhesion on the PHBHV/LDH-SDS 5% substrate**.**

Additionally, actin microfilament formation may be assigned with a shape modeling process that occurs in response to the direct contact of the cell with the biomaterial. The actin cytoskeleton underlies the cell adhesion process, which is highly important for further tissue formation. These results are in agreement with the AFM observations. As shown in Figure 9, hASCs were able to adhere and to display an elongated morphology on the PHBHV/LDH-SDS 5% nanocomposite biomaterial.

### 3.6. hASCs Viability in Contact with PHBHV, PHBHV/LDH-SDS 1%, PHBHV/LDH-SDS 3%, and PHBHV/LDH-SDS 5% Biomaterials

**Figure 10:**
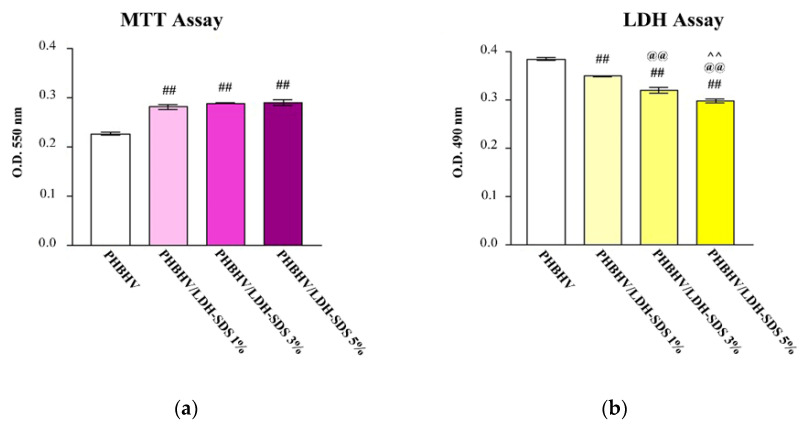
Quantification of (**a**) hASCs’ viability on PHBHV, PHBHV/LDH-SDS 1%, PHBHV/LDH-SDS 3%, and PHBHV/LDH-SDS 5% biomaterials, as revealed by MTT assay at 24 h post-seeding, and (**b**) PHBHV, PHBHV/LDH-SDS 1%, PHBHV/LDH-SDS 3%, and PHBHV/LDH-SDS 5% biomaterials’ cytotoxic potential on hASCs after 24 h of culture, as revealed by the lactate dehydrogenase assay. “##”: *p* < 0.001 (hASCs/PHBHVvs.hASCs/PHBHV/LDH-SDS 1%, hASCs/PHBHV/LDH-SDS 3%, and hASCs/PHBHV/LDH-SDS 5% biohybrids); “@@”: *p* < 0.001 (hASCs/PHBHV/LDH-SDS 1% vs.hASCs/PHBHV/LDH-SDS 3%, and hASCs/PHBHV/LDH-SDS 5%); **“**^^”: *p* < 0.001 (hASCs/PHBHV/LDH-SDS 3% versus hASCs/PHBHV/LDH-SDS 5%).

MTT assay: hASCs’ viability on the PHBHV, PHBHV/LDH-SDS 1%, PHBHV/LDH-SDS 3%, and PHBHV/LDH-SDS 5% biomaterials was quantitatively determined by an MTT spectrophotometric assay. In this context, the hASCs/PHBHV, hASCs/PHBHV/LDH-SDS 1%, hASCs/PHBHV/LDH-SDS 3%, and hASCs/PHBHV/LDH-SDS 5% biohybrids were subjected to MTT spectrophotometric assay after 24 h of culture, and the results are represented in Figure 10a.

Our results showed that hASCs survived on PHBHV, PHBHV/LDH-SDS 1%, PHBHV/LDH-SDS 3%, and PHBHV/LDH-SDS 5% nanocomposites after 24 h of culture, but significant differences were observed when comparing the hASCs’ viability on PHBHV with the hASCs’ viability on the PHBHV/LDH-SDS 1%, PHBHV/LDH-SDS 3%, and PHBHV/LDH-SDS 5% biomaterials (*p* < 0.001). No significant differences were determined between hASCs/PHBHV/LDH-SDS 1%, hASCs/PHBHV/LDH-SDS 3%, and hASCs/PHBHV/LDH-SDS 5% (*p* > 0.05).

### 3.7. Biomaterials’ Cytotoxic Potential on hASCs

The cytotoxic potential of the PHBHV, PHBHV/LDH-SDS 1%, PHBHV/LDH-SDS 3%, and PHBHV/LDH-SDS 5% nanocomposites was evaluated by spectrophotometric quantification of the lactate dehydrogenase enzyme release in the culture media through hASCs seeded in direct contact with the samples (Figure 10b). The lactate dehydrogenase levels detected in the culture medium harvested from the hASCs/PHBHV were found to be statistically significant lower (*p* < 0.001) compared with the results obtained for the PHBHV/LDH-SDS 1%, PHBHV/LDH-SDS 3%, and PHBHV/LDH-SDS 5% biohybrids, showing that the cell death rate on these biomaterials was significantly lower than that in contact with the PHBHV sample Additionally, the same significant differences (*p* < 0.001) were detected between all of the biohybrids.

## 4. Conclusions

PHBHV/LDH-SDS nanocomposites with a disorderly exfoliation/intercalation structure were successfully obtained by melt mixing with modified mineral clay. The exfoliation/intercalation structure is revealed by XRD and TEM results. An important key role in the exfoliation/intercalation process is played by the modification of mineral clay with an organic anion by increasing compatibility of the two phases. It was shown that the exfoliation/intercalation process was achieved only by adding a larger amount of modified mineral clay (5%). An exfoliated/intercalated nanocomposite led to increased mechanical properties due to the disorderly clay layer arrangement. The nanocomposite materials obtained here have the following potential biomedical applications: as dressings; tissue engineering due to properties such as biocompatibility, biodegradability, and production from renewable resources of the natural polyester; and the ability for wound healing of the mineral clay.

The fluorescent labeling of actin filaments showed that hASCs displayed a normal morphology in contact with all of the nanocomposite materials. However, the spectrophotometric assays revealed significant differences between the samples in terms of cellular viability and biomaterials’ cytotoxic potential. The MTT assay results indicate that hASCs seeded on PHBHV/LDH-SDS 1%, PHBHV/LDH-SDS 3%, and PHBHV/LDH-SDS 5% displayed the best viability rate, whereas the lactate dehydrogenase assay results showed that the PHBHV/LDH-SDS 5% biomaterial displayed the lowest cytotoxic effect on hASCs. In conclusion, the PHBHV/LDH-SDS 5% biomaterial could be considered for further in vivo studies on animal models.
